# Genotype I of Japanese Encephalitis Virus Virus-like Particles Elicit Sterilizing Immunity against Genotype I and III Viral Challenge in Swine

**DOI:** 10.1038/s41598-018-25596-1

**Published:** 2018-05-10

**Authors:** Yi-Chin Fan, Jo-Mei Chen, Jen-Wei Lin, Yi-Ying Chen, Guan-Hong Wu, Kuan-Hsuan Su, Ming-Tang Chiou, Shang-Rung Wu, Ji-Hang Yin, Jiunn-Wang Liao, Gwong-Jen J. Chang, Shyan-Song Chiou

**Affiliations:** 1 Graduate Institute of Microbiology and Public Health, National Chung Hsing University, Taichung, Taiwan; 2 Department of Veterinary Medicine, National Pingtung University of Science and Technology, Pingtung, Taiwan; 3 Institute of Oral Medicine, College of Medicine, National Cheng Kung University, Tainan, Taiwan; 4 Graduate Institute of Veterinary Pathobiology, National Chung Hsing University, Taichung, Taiwan; 5 Arboviral Diseases Branch, Center for Disease Control and Prevention, Fort Collins, Colorado, United States of America

## Abstract

Swine are a critical amplifying host involved in human Japanese encephalitis (JE) outbreaks. Cross-genotypic immunogenicity and sterile protection are important for the current genotype III (GIII) virus-derived vaccines in swine, especially now that emerging genotype I (GI) JE virus (JEV) has replaced GIII virus as the dominant strain. Herein, we aimed to develop a system to generate GI JEV virus-like particles (VLPs) and evaluate the immunogenicity and protection of the GI vaccine candidate in mice and specific pathogen-free swine. A CHO-heparan sulfate-deficient (CHO-HS(-)) cell clone, named 51-10 clone, stably expressing GI-JEV VLP was selected and continually secreted GI VLPs without signs of cell fusion. 51-10 VLPs formed a homogeneously empty-particle morphology and exhibited similar antigenic activity as GI virus. GI VLP-immunized mice showed balanced cross-neutralizing antibody titers against GI to GIV viruses (50% focus-reduction micro-neutralization assay titers 71 to 240) as well as potent protection against GI or GIII virus infection. GI VLP-immunized swine challenged with GI or GIII viruses showed no fever, viremia, or viral RNA in tonsils, lymph nodes, and brains as compared with phosphate buffered saline-immunized swine. We thus conclude GI VLPs can provide sterile protection against GI and GIII viruses in swine.

## Introduction

Japanese encephalitis virus (JEV) is maintained in the transmission cycle between amplifying hosts and *Culex* mosquito vectors^1^ or in a vector-free manner between pigs^2^. JEVs are classified into five phylogenetically distinctive genotypes (GI-GV)^3^. Historically, the GIII virus was the dominant genotype in JEV epidemic regions; however, the emerging GI virus has gradually replaced the GIII virus and has become the dominant genotype in Eastern and Southeastern Asian countries since the 1990s^4^. The mosquito-bird cycle maintains the virus, infection of swine may bring the virus into contact with humans, and humans and horses are dead-end hosts in endemic regions^5–7^. Although JEV infection in adult pigs is usually asymptomatic, there is an increase in morbidity and mortality in juvenile animals, and infection of pregnant sows can cause abortion and stillbirth^8^.

Implementation of JE vaccination has successfully reduced the annual human JE cases in many countries of Asia^9^ and reduced the rate of abortion and stillbirth in commercial pig farms^10^. Vaccinating pigs is expected to suppress the viral transmission and reduce JEV infection in humans^11–13^. However, vaccination has only applied to sows to prevent abortion rather than to block viral circulation, and a high seroconversion rate is consistently detected in pig farms^14–16^.

The current JE vaccines for humans or domestic animals are derived from GIII viruses, with amino acid sequences on the E protein significantly different from those in the GI virus^17^. Several studies have focused on vaccine efficacy affected by genotype replacement. Overall results suggested that the GIII JEV vaccine might temporarily protect against GI virus infection, especially for travelers, but vaccine efficacy for long-term protection might be reduced in GI JEV epidemic or endemic countries or regions^18–25^.

Considerations of a next-generation JEV vaccine for sows might include an ability to block virus transmission and induce cross-protective activity against the currently dominant GI virus and other genotypic viruses, especially the co-circulating GIII virus in some JEV endemic regions^18,26,27^. Non-infectious and self-assembled virus-like particles (VLPs) can elicit protective immunity against viral infection and are a suitable vaccine candidate for many viruses including JEV^28–33^. Therefore, we developed GI JEV VLPs that were continually produced from the stable clone and evaluated the antibody response and cross-protective potency against GI through GIV viruses in VLP-immunized mice and SPF swine. GI JEV VLPs elicited antibodies cross-neutralizing GI through GIV JEV and cross-protected mice and special pathogen-free (SPF) pigs against GI and GIII JEV infection. The sterile protection observed in pigs implied a potential for GI VLPs protection against abortion and blocking JEV transmission in the pig farm.

## Results

### Characterization of GI JEV VLPs produced from the 51-10 clone

**Figure 1:**
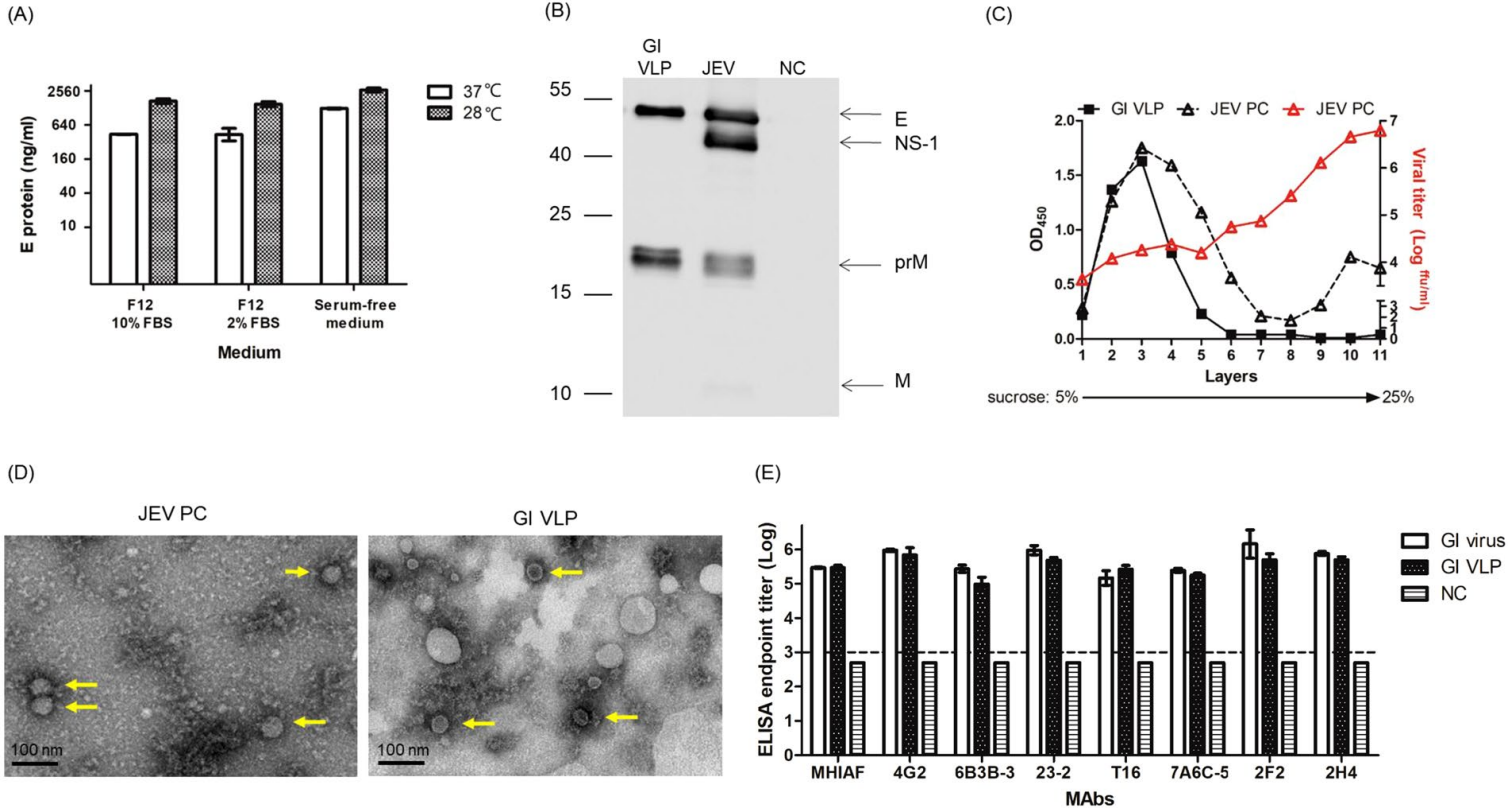
Characterization of the GI JEV VLPs produced from the stable 51-10 clone. (**A**) The yield of VLPs was determined in the supernatant of the 51-10 clone cultured in F12 medium supplemented with 2% or 10% FBS or in serum-free medium at 37 °C or 28 °C for 3 days by Ag-ELISA. (**B**) The secreted GI JEV VLP antigens collected from the supernatant of the 51-10 clone were analyzed by Western blot assay with anti-JEV HIAF and are indicated as GI VLPs. The supernatant collected from GI JE YL2009–4 virus-infected or the non-infected cells is presented as JEV or NC (negative control). Full-length Western blots are presented in Supplementary Fig. S2. (**C**) The GI YL2009-4 virus (JEV PC) and 51-10 clone-derived VLPs underwent ultracentrifugation in a gradient of 5% to 25% sucrose. The distribution of JEV protein (Δ) and infectious activity () of JEV PC in the gradient was examined by Ag-ELISA and micro-plaque assay, respectively. The distribution of GI VLP particles (■) was detected by Ag-ELISA. (**D**) The morphology of purified GI JEV YL2009-4 virions (JEV PC) and GI VLP by staining with 2% uranyl acetate was analyzed by transmission electron microscopy. (**E**) The antigenicity of GI VLP was analyzed by Ag-ELISA with mouse anti-JEV HIAF (MHIAF) and flavivirus-reactive mAbs (4G2, 6B3B-3, 23-2, T16, 7A6C-5, 2F2, and 2H4). The supernatant collected from non-transfected CHO-HS(-) cells was used as the NC (negative control). The endpoint titers between GI virus and GI VLPs were run in duplicates and calculated by Student *t* test. Data are mean ± SD.

We constructed and characterized the GI VLP expressing plasmids (Supplementary Methods, Supplementary Figs S1 and S2 in Supplementary information) and established the CHO-HS(-) cell-derived 51-10 clone that stably secreted GI VLP antigens (Supplementary Methods, Supplementary Figs S3 and S4 in Supplementary information). We optimized the culture condition and propagated the 51-10 clone in serum-free media at 28 °C with the VLP yield at 2614.8 ng/ml (Fig. 1A). The viral E, NS1, prM, and M proteins were detectable in the JEV cultured sample, and the same size of E and prM proteins appeared in the 51-10 clone produced VLPs (Fig. 1B). The concentrated GI VLPs were analyzed by rate zonal centrifugation using 5% to 25% sucrose gradient (Fig. 1C). The Vero-derived GI JEV, used as a positive control (JEV PC), formed two OD_450_ peaks in the gradient. The higher density OD_450_ peak at the bottom of the gradient showed the highest infectious viral titers. The broad lower density OD_450_ peak seemed to be VLPs because they exhibited low viral titers, slow sedimentation, and distributed from the 2^nd^ to 4^th^ gradient factions, where the GI VLP sample was located. The morphology of GI VLPs was verified using transmissible electron microscope (TEM, Fig. 1D). We observed empty particles of 30 to 40 nm in diameter in the GI VLP-purified sample in contrast to solid particles of about 50 nm in diameter in the infectious GI JEV sample. Total protein of the GI VLP-purified sample was examined by SDS page (Supplementary Fig. S5). In addition, we analyzed the antigenicity of the viral E protein organized on VLPs with a panel of monoclonal antibodies (mAbs) including the flavivirus group (4G2, 6B3B-3, and 23–2) and complex cross-reactive (T16 and 7A6C-5), and JEV type-specific (2F2 and 2H4) mAbs by the antigen-capture enzyme-linked immunosorbent assay (Ag-ELISA). The supernatant collected from non-transfected CHO-HS(-) cells (NC) were not reactive to the mAbs tested. Antigens derived from GI VLPs or GI virion particles showed the similar Ag-ELISA end-point titers against all mAbs with no statistically significant differences between them (Fig. 1E). These results indicated that GI VLPs derived from the 51-10 clone formed a homogeneous empty-particle morphology and exhibited similar antigenic activity as GI virions.

### Antibody response elicited by GI JEV VLPs in BALB/c mice

**Figure 2:**
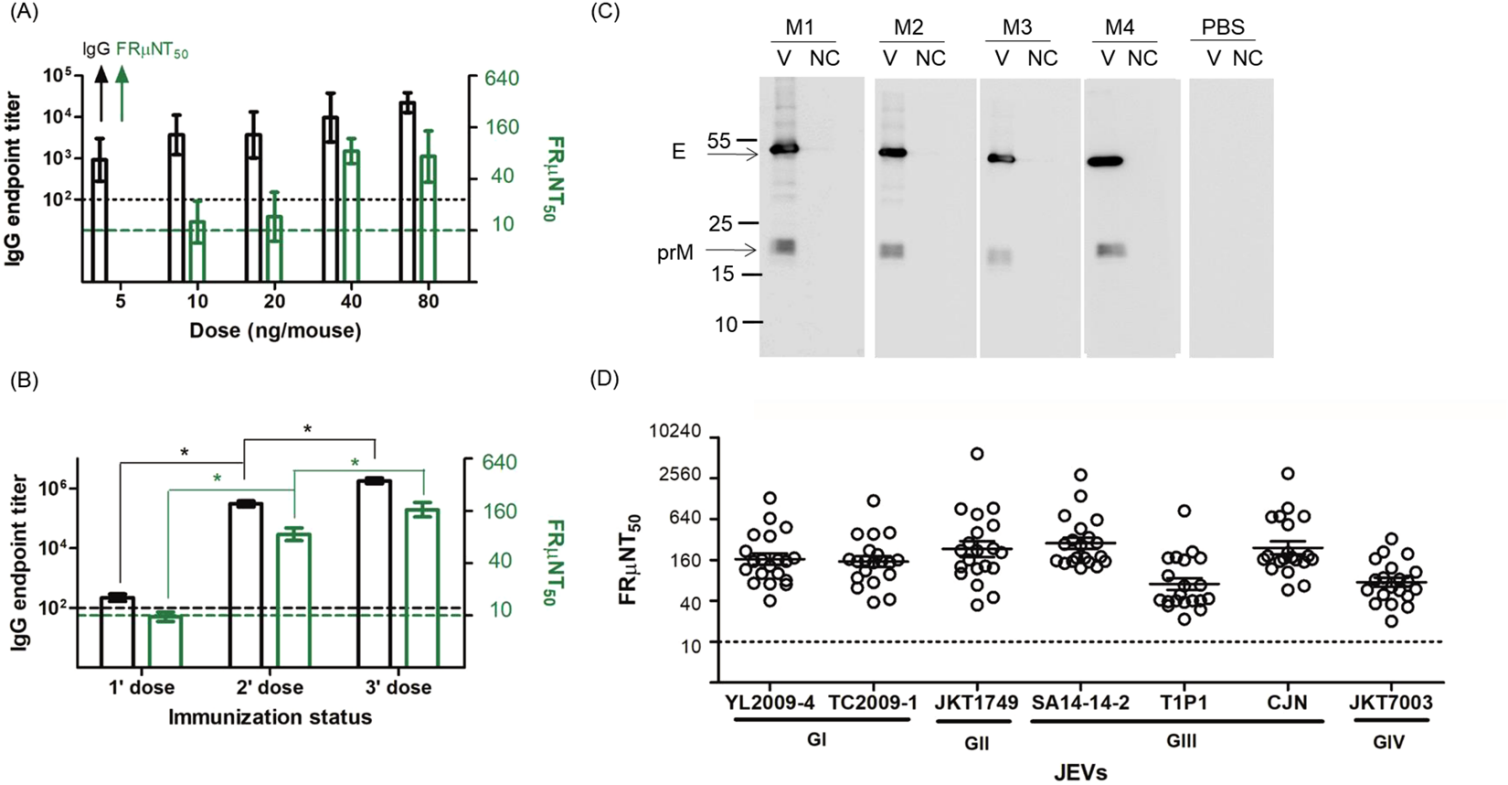
Immunogenicity of GI JEV VLPs in mice. (**A**) BALB/c mice (5 mice each group) received 2 doses of 5, 10, 20, 40, or 80 ng GI VLPs subcutaneously, then the IgG antibody (black, left y-axis) and neutralizing activity (green, right y-axis) were measured by mouse IgG antibody capture (GAC)-ELISA and focus-reduction micro-neutralization (FRμNT) assay. (**B**) 20 BALB/c mice were vaccinated with 1, 2, or 3 doses of 40 ng GI VLPs, and the endpoint titers of IgG antibodies against VLPs (black, left y-axis) and homologous neutralizing activity (green, right y-axis) were estimated by mouse GAC-ELISA and FRμNT assay. (**C**) The reactivity of IgG antibodies against GI YL2009-4 virus proteins (V) and the cellular supernatant (NC; negative control) among 3-dose PBS- or GI VLP-immunized mice (M1 to M4) were analyzed by Western blot assay. Full-length Western blots are presented in Supplementary Fig. S6. (**D**) The heterologous neutralizing antibodies elicited by 3 doses of GI VLP against GI, GII, GIII, and GIV JEVs were determined by FRμNT_50_. Data are mean ± SD compared by Student *t* test, one-way ANOVA, or post-test with Turkey’s Multiple Comparison Test. *P < 0.05.

The immunogenicity of JEV vaccine is usually evaluated in the mouse model and neutralization (Nt) antibody titers higher or equal to 10 of 50% focus-reduction micro-neutralization titer (FRμNT_50_) have been accepted as the protective threshold^34^. BALB/c mice were subcutaneously (sc) immunized with two doses of 5, 10, 20, 40, or 80 ng GI VLPs mixed with Freund’s incomplete adjuvant at age 6 and 8 weeks. All immunized mice elicited a total IgG antibody titer which ranged from 10^2.96^ to 10^4.35^ and the dose–response escalation of Nt antibodies ranged from <10 to 84. Mice receiving 40 ng GI VLPs showed the highest neutralizing antibodies but with no statistical significance (P > 0.05) (Fig. 2A). In the second immunogenicity study, groups of BALB/c mice (twenty per group) were vaccinated with one, two, or three doses of 40 ng GI VLPs. The total IgG titers in pooled serum specimens drastically increased after each boost immunization, with IgG titers estimated at 10^6.21^ after the third booster. The homologous neutralizing activity against GI YL2009–4 virus dose-dependently increased, with GMT FRμNT_50_ titers of 144 after 3-dose vaccination (Fig. 2B). The Western blot showed that antibodies elicited by GI VLP vaccination only recognized viral E and prM proteins (Fig. 2C). The Nt antibodies elicited by the 3-dose regiment of GI VLPs also neutralized GI, GII, GIII, and GIV JEVs, with the highest GMT FRμNT_50_ titers of 229 against GIII SA14-14-2 virus and the lowest of 59 against GIII T1P1 virus (Fig. 2D). Hence, three-dose (40 ng/dose) sc vaccination was efficient to induce an antibody response recognizing viral E and prM proteins and cross-neutralizing GI through GIV JEVs.

### Cross-protection against GI and GIII viruses in GI VLP-immunized mice

The protective efficacy of the JEV vaccine was also evaluated in the mouse challenge model, which exhibited neurological symptoms after intracranial (ic) viral infection. Mice were sc vaccinated with 3 doses of 40 ng GI VLPs, as indicated in the previous section. Mice were grouped and ic challenged with 100LD_50_ of GI YL2009-4 or GIII T1P1 virus, and the cumulative survival curve of mice was recorded until 21 days post-inoculation (DPI) (Table 1). Regardless of GI or GIII virus infection, PBS control mice showed neurological symptoms at 6 DPI and eventually were killed at predefined humane endpoints as approved by 20% weight loss at 9 DPI (see Methods). GI VLP-immunized mice with Nt antibodies from 34 to 1282 all survived the GI virus infection and 9/10 (90%) of the immunized mice with Nt titers from 10 to 824 survived the GIII virus infection. Thus, the GI VLP vaccine provided the mice with a high potency protection against homologous or heterologous JEVs.

**Table 1:** Protection of GI JEV VLP vaccine against GI and GIII JEV infection in mice.

Immunogens	JEV challenge (genotype)	FRμNT_50_ titers against JEV challenge	No. of surviving mice/total	Protective rate^a^ (%) (surviving mice/total)	Average time to die (ATD)
GI VLP	YL2009-4 (I)	<10	0	100* (10/10)	—
		10–<40	1/1		
		40–<160	6/6		
		≥160	3/3		
	T1P1 (III)	<10	0	90* (9/10)	9
		10–<40	2/2		
		40–<160	5/6		
		≥160	2/2		
PBS	YL2009-4 (I)	<10	0/8	0 (0/8)	9
	T1P1 (III)	<10	0/8	0 (0/8)	8.5

^a^Data was compared by Student t test. *P < 0.05.

### Antibody response elicited by GI VLP vaccine in SPF swine

**Figure 3:**
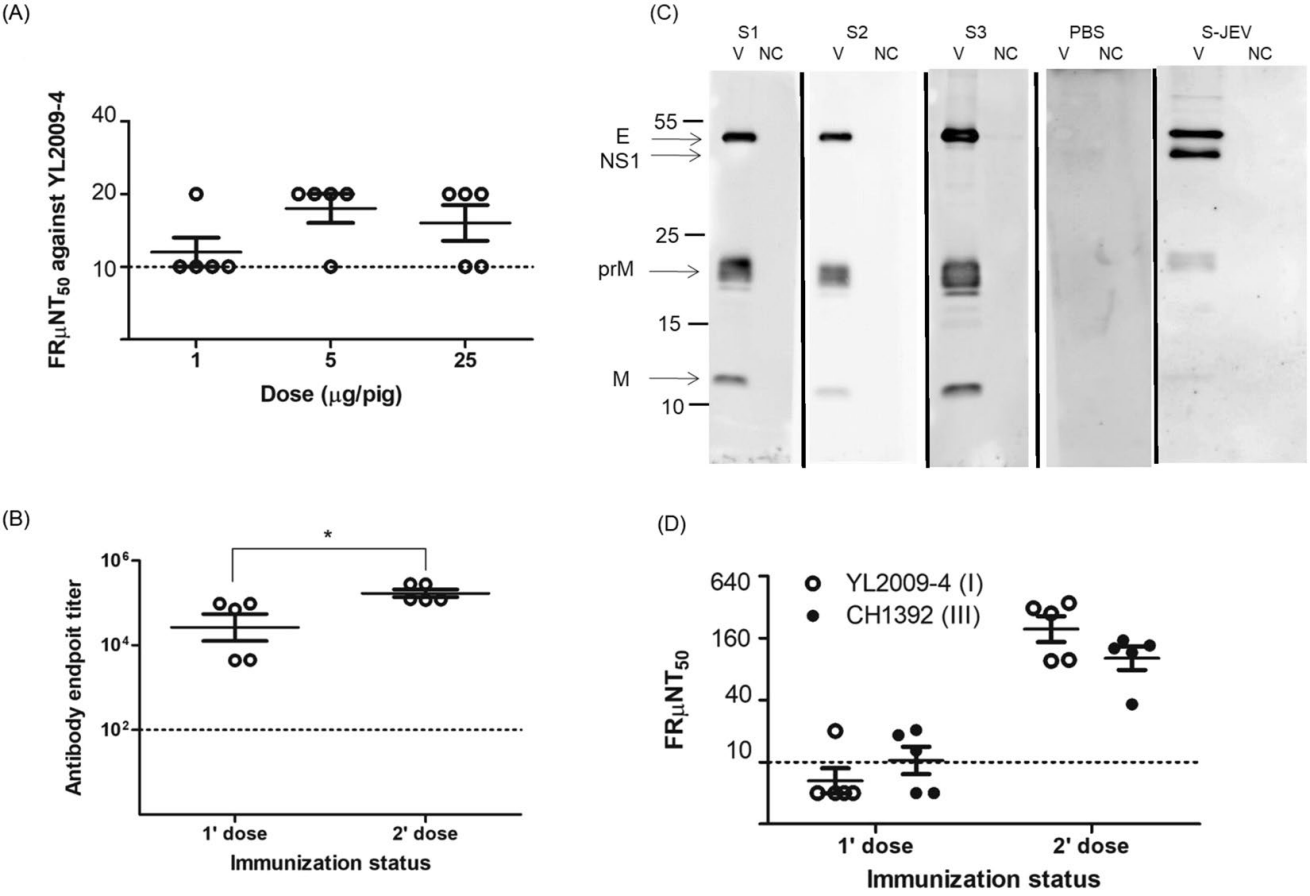
Immunogenicity of GI JEV VLPs in SPF swine. (**A**) Second-generation SPF swine (5 pigs each group) were inoculated with 1, 5, or 25 μg GI VLPs with ISA201-adjuvant, and neutralizing antibodies in the serum specimens were determined by FRμNT_50_. (**B**) SPF pigs (5 pigs each group) were immunized with 1 or 2 doses of 5 μg GI VLP, and the antibody response was detected by swine Ag-ELISA. (**C**) The reactivity of antibodies against GI YL2009-4 virus proteins (V) and the cellular supernatant (NC) among 2-dose PBS- or GI VLP-immunized SPF swine (S1 to S3) was analyzed by western blot assay. The serum plasma collected from naturally infected swine (S-JEV) was a positive control. Full-length Western blots are presented in Supplementary Fig. S7. (**D**) SPF swine (5 pigs each group) were immunized with 1 or 2 doses of 5 μg GI VLPs, and the heterologous neutralizing activity against GI and GIII viruses was determined by FRμNT_50_. Data are mean ± SD and were compared by Student *t* test. *P < 0.05.

We inoculated second-generation SPF swine (5 pigs in each group) with various doses of GI VLPs to determine the optimum dosage for eliciting Nt antibodies. The FRμNT_50_ was determined individually from groups, and the GMT titer of 12, 17, or 15 was shown for swine receiving a single immunization of 1 μg, 5 μg, or 25 μg GI VLPs, respectively (Fig. 3A). Then swine were vaccinated with 1 or 2 doses of 5 μg GI VLPs. The endpoint IgG titers of anti-GI VLP antibodies was 10^4.42^ after the primary immunization and the titers were significantly elevated to 10^5.23^ after a booster (Fig. 3B). Serum specimens from GI VLP immunized pigs, similar to naturally infected swine, elicited antibodies recognizing E, prM and M proteins. The naturally infected swine serum also clearly recognized NS1 protein but serum from swine immunized with VLP did not (Fig. 3C). 60% (3/5) of pigs elicited Nt antibodies after primary immunization. Four weeks after the second immunization all immunized pigs elicited potent Nt antibodies with the GMT FRμNT_50_ titers of 194 and 101 against GI and GIII viruses, respectively (Fig. 3D). We concluded that 2 doses at 5 μg/dose of GI VLPs in SPF pigs is sufficient to elicit antibodies recognizing E, prM, and M proteins and neutralizing homologous GI and heterologous GIII JEVs.

### Cross-protection against GI and GIII viruses in GI VLP-immunized swine

The SPF swine received 2 doses of 5 μg/dose of GI VLPs or PBS control and were subcutaneously challenged with 10^7^ FFU of GI YL2009-4 or GIII CH1392 viruses (Table 2). The PBS-control pigs rapidly developed serum viremia, peaked at 2-day post inoculation (DPI), with 10^2.5^ to 10^4.7^ (ffu/ml) of the infectious viruses and 10^5.1^ to 10^7.1^ (copies/ml) of viral RNA; all had an increase in body temperature of 39.5 °C to 41.1 °C by 3 DPI. In contrast, GI VLP-immunized swine had normal body temperature (37.8 °C to 38.5 °C) and regardless of the challenging strains, remained healthy with undetectable viremia and viral RNA in serum before being euthanized at 8-DPI. We further confirmed, similar to a previous report^2^, that viral RNAs were detected in tonsils, lymph nodes, and brains of all PBS control pigs. At 8-DPI, tonsils, lymph nodes, and brain harvested from PBS-control pigs showed 10^5.6^ to 10^7.2^, 10^4.2^ to 10^7.0^, and 10^4.5^ to 10^5.8^ (copies/g) viral RNA, respectively. None of the GI VLP-immunized swine had detectable levels of viral RNA in any of the tissues tested. PBS-control, viral challenged pig brains showed multifocal perivascular cuffing and multifocal gliosis (Supplementary Fig. 8A and B). In contrast, regardless of difference in GI or GIII virus used for challenge, GI VLP-immunized and virus challenged pig brains showed no observable lesions in the parenchyma or vessels (Supplementary Fig. 8C and D). PBS-control challenged pigs showed a great increase in Nt antibody titers, but only 0.3- to 9.3-fold increases in Nt titers among GI VLP-immunized swine after viral challenge (Table 2). This data suggests the protective immunity elicited in GI VLP immunization in swine can either drastically or completely suppress JEV viral replication to undetectable levels by all measures, thus providing sterile protection against both GI and GIII viral infections.

**Table 2:** GI JEV VLP-vaccinated pigs challenged with GI and GIII viruses.

Immunogens	JEV challenge	Viremia^a^		Tonsils^b^	Lymph nodes	Brain	Body temperature^c^	Fold increase in FRμNT_50_^d^
		Viral titers (ffu/ml) (mean ± SD)	Viral RNA (copies/ml) (mean ± SD)	Viral RNA (copies/g) (mean ± SD)				
PBS	YL2009-4	3.6 ± 0.1	6.3 ± 0.5	7.1 ± 0.3	6.1 ± 0.2	5.0 ± 0.4	40.7	1093.0*
PBS	YL2009-4	3.4 ± 0.1	5.6 ± 0.3	6.3 ± 1.2	4.5 ± 0.1	5.2 ± 0.4	40.8	938.0*
PBS	YL2009-4	4.6 ± 0.2	7.1 ± 0.2	6.9 ± 0.1	7.0 ± 0.2	5.8 ± 0.3	41.1	1966.6*
PBS	YL2009-4	4.7 ± 0.2	6.3 ± 0.3	7.2 ± 0.5	6.7 ± 0.3	5.1 ± 0.1	40.7	1698.2*
PBS	CH1392	2.8 ± 0.2	5.8 ± 0.2	5.6 ± 1.3	4.2 ± 0.1	4.5 ± 0.5	40.2	2048.0*
PBS	CH1392	2.5 ± 0.2	5.1 ± 0.1	6.1 ± 0.1	4.5 ± 0.1	4.6 ± 0.3	39.8	1220.6*
PBS	CH1392	2.5 ± 0.3	5.6 ± 0.4	6.2 ± 0.4	4.8 ± 0.2	5.1 ± 0.1	39.9	814.8*
PBS	CH1392	2.8 ± 0.2	5.8 ± 0.1	6.6 ± 0.2	5.2 ± 0.1	5.2 ± 0.2	39.5	1367.0*
GI VLP	YL2009-4	<1.3	<2.0	<2.0	<2.0	<2.0	38.3	0.8
GI VLP	YL2009-4	<1.3	<2.0	<2.0	<2.0	<2.0	38.4	3.6
GI VLP	YL2009-4	<1.3	<2.0	<2.0	<2.0	<2.0	38.1	1.6
GI VLP	YL2009-4	<1.3	<2.0	<2.0	<2.0	<2.0	38.5	9.3*
GI VLP	CH1392	<1.3	<2.0	<2.0	<2.0	<2.0	37.8	0.4
GI VLP	CH1392	<1.3	<2.0	<2.0	<2.0	<2.0	37.9	0.3
GI VLP	CH1392	<1.3	<2.0	<2.0	<2.0	<2.0	38.2	1.3
GI VLP	CH1392	<1.3	<2.0	<2.0	<2.0	<2.0	37.9	3.3
GI VLP	CH1392	<1.3	<2.0	<2.0	<2.0	<2.0	38.5	6.6*

^a^The viral titers and viral RNA in pig plasma was detected and duplicated by micro-plaque assay and real-time RT-PCR at 2 DPI. ^b^The viral RNA was quantified in duplicate samples of tonsils, lymph nodes, and brain from infected pig at 8 DPI by real-time RT-PCR. ^c^The highest body temperature of swine was indicated during 8 days post-challenge. ^d^In comparing FRμNT_50_ at 8 DPI to the value before challenge, a fold increase was indicated and a ≥4-fold increase was noted by an asterisk.

## Discussion

JEV vaccination is the most effective way to prevent epidemics in humans and pigs^9,10^. The safety of using a live-attenuated JEV vaccine in pregnant sows remains uncertain^35^, and formalin inactivation has been shown to alter the antigenic structure of E protein^36^. Despite potential problems with existing vaccines, they are effective at protecting animals from disease^37^. A non-infectious JEV vaccine could be a suitable candidate especially with VLPs, which are considered better than subunit E proteins and peptides in eliciting conformational-dependent neutralizing antibodies^38^. The recombinant flaviviral VLPs form a smaller sized particle than the virions but maintain similar antigenicity and protect mice against viral infection^39–43^. The cryo-EM structure of the TBEV VLPs incorporate 30 E dimers in T = 1 icosahedral symmetry in contrast to the T = 3 arrangement for the virions^44^. In this study, GI JEV VLPs formed a smaller size of particle as compared to infectious virions but exhibited the same mAb binding profile as GI virions. Importantly, highly potent neutralizing antibodies were elicited in GI VLP immunized mice and pigs.

Several GIII JEV VLP-expressing plasmids and stable-expressing cell lines, including mammalian and insect cell lines, have been established but encountered problems such as low secretion of VLP, the fusogenic nature of the stable cell lines, and contaminants of extracellular vesicles^45–48^. Here, we increased the secretion of GI JEV VLP by utilizing the plasmid encoding the modified JEV signal peptide to ensure the secretion of VLP and by culturing the CHO-HS(-)-derived 51-10 stable clone in serum free media at 28 °C as shown in the previous study^49^. The baculovirus-insect cell system showed an enhancement in the production of GIII JEV VLP as compared to the recombinant CHO-K1 cell system^45^. To reduce the production cost the insect cell system might be considered for the production of GI JEV VLP in the future. The 51-10 clone was derived from CHO-HS(-) cells. This cell line is a low-susceptible cell line for JEV infection^50^. The 51-10 clone stably expressing GI JEV VLP exhibited a normal cellular morphology without the detection of cell fusion. Kojima *et al*. observed a similar result by utilizing a low-susceptible cell line of JEV to stably produce GIII JEV VLP^51^.

The anti-JEV neutralizing antibodies primarily recognize E protein^52^. E amino acid sequences are different among five genotypes of JEV. Amino acid substitutions at positions 222 and 327 in the E protein of GI virus might be critically related to the immunogenicity of vaccine^53^. The neutralizing antibody titer was lower against GI virus than GIII virus: 5.9- to 10.2-fold lower titers among vaccinees receiving GIII-inactivated vaccines^22,23^, 2.1-fold lower titers among vaccinees receiving GIII live-attenuated vaccine^19^, and 14.6-fold lower titers among swine receiving GIII live-attenuated vaccine^20^. Here, the neutralizing antibody titer elicited by GI VLP vaccine was higher against GI than GIII virus by 1.2–fold (157 vs 130; Fig. 2D) in mice and 1.9-fold (194 vs 101; Fig. 3D) in pigs. Two GI JEV vaccine candidates, inactivated and recombinant pseudorabies virus expressing GI JEV VLPs, exhibited high homologous neutralizing activity against GI virus but were untested against GIII virus in mouse and pig models^54,55^. Here we showed that our GI VLPs exhibited high immunogenicity against GI virus in mice and also potent neutralizing antibodies against GII, GIII, and GIV viruses. This vaccine candidate also elicits cross-neutralizing antibodies and cross-protection against GI and GIII viral challenge in swine. Thus, compared to existing GIII vaccines, our GI VLP vaccine elicits broaden cross-neutralizing antibodies against GI, GII, GIII, and GIV JEVs, which share 94%-99% homology in amino acid sequences of E protein. The limitations of this study is that we were not able to secure and include GV virus in the study. GV virus has only been isolated from mosquitoes, and has not been isolated in swine or other vertebrate hosts^56,57^ since the first isolate was identified from a human patient brain in 1952^58^.

The JEV-challenged swine model we established exhibited comparable viremic and viral distribution in tissues of the central nervous and lymphatic systems as showed in a previous study^2^. Previous research indicated that swine vaccinated with lentiviral GIII JEV prM/E vector were not fully protected because viral RNA was detected in the central nervous system and lymphoid tissues after homologous viral challenge^59^. Our study is the first to evaluate the cross-neutralizing activity and cross-protection potency of a GI JEV-derived vaccine in swine. We found no detectable viremia and viral RNA in tonsils, lymph nodes, and brain specimens from GI VLP-immunized pigs regardless of GI or GIII viral challenge. Tonsils have been especially indicated as the possible source of persistent infection and replication to transmit JEV via contact or the oronasal route^2^. In addition, the neutralizing antibody titers were only slightly increased among GI VLP-vaccinated pigs after GI or GIII virus challenge (Table 2). Thus, the GI JEV VLP vaccine may provide full cross-protection against the dominant GI virus and co-circulating GIII virus in JE-epidemic areas of Asia^26,27^. We did not assess the duration of immunity and protection since the GI VLP-vaccinated swine were challenged with virus 4 weeks after the final immunization.

The major indication for the use of JEV vaccination in pigs is for the prevention of abortion and stillbirth in pregnant sows and for reducing the JEV epidemic in pigs and human^37^. To reduce economic costs of JEV infection in pigs and ultimately reduce the impact of JEV in humans requires preventing JEV circulation in pigs. This can be accomplished by implementing a non-infectious JE vaccination campaign for all pigs and reducing the viral spread from pigs to other hosts or mosquitos. The sterile protection of GIII JEV vaccines against JEV infection has not previously been addressed in swine^10^. Here, the GI JEV VLPs provided sterilizing immunity against GI and GIII JEV infection in pigs. Thus, our vaccine preparation has the potential to reduce loss of income due to abortion/stillbirth in the swine industry and also capability of decreasing JEV circulation in humans and pigs by blocking mosquito-borne or mosquito-free transmission. As compared with the GIII JEV vaccine, the GI JEV VLP vaccine cost is expected to be lower by increasing production scale using serum-free, chemically-defined media for the production of VLPs in CHO-HS(-) cell clones. By reducing the vaccine cost and improving the coverage of JEV vaccination in pigs, we might be able to realize the economic benefit of reducing the abortion rate and blocking JEV circulation in pig farms.

## Methods

### Ethics Statement

Female BALB/c mice were purchased from the National Laboratory Animal Center of Taiwan, certified by an Association for Assessment and Accreditation of Laboratory Animal Care (AAALAC), and raised in the animal facility of National Chung Hsing University (NCHU), Taiwan. Second-generation and specific pathogen-free (SPF) swine were purchased from the Agricultural Science and Technology Research Institute of Taiwan and housed in a negative air-pressure animal facility certified by the AAALAC in the Animal Disease Diagnostic Center of National Pingtung University of Science and Technology (NPUST), Taiwan. Mouse and swine experiments followed protocols approved by the Institutional Animal Care and Use Committee (IACUC) of NCHU (Approval No: 101–88) and NPUST (NPUST-104-013) and were carried out in accordance with the Guide for the Care and Use of Laboratory Animals (NRC 2011).

### Cell lines, viruses, and antibodies

COS-1 and VERO cells (a kind gift of Dr. GJ Chang, US Centers for Disease Control and Prevention, Fort Collins, CO) were cultured in Dulbeco’s Modified Eagle’s Minimal Essential medium (DMEM, Gibco) supplemented with 10% and 5% heat-inactivated fetal bovine serum (FBS, Gibco), respectively. Heparan sulfate is a critical cellular attachment factor for initiating JEV infection^50^. CHO-pgsA745 (CHO-HS(-), a kind gift of Dr. WJ Chen, Chang Gung University, Taiwan), deficient in heparan sulfate expression, was used to select stably expressed JEV VLPs. CHO-HS(-) were cultured in Ham’s F12 medium (F12, Gibco) containing 10% heat-inactivated FBS.

In this study, we used four genotypes of JEVs, including GI YL2009-4 strain (JF499789.1), GI TC2009-1 strain (JF499790.1), GIII SA14-14-2 strain (AF315119.1), GIII T1P1 strain (AF254453.1), GIII CH1392 strain (AF254452.1), GIII CJN strain (AY303793.1 and AY303794.1), GII JKT1749 strain (U70405.1), and GIV JKT7003 strain (U70408.1). Mouse anti-JEV hyperimmune ascitic fluid (HIAF) and rabbit anti-JEV polyclones were kindly provided by Dr. GJ Chang. We characterized the antigenicity of JEV and VLP by a panel of the flavivirus-specific monoclonal antibodies (mAbs) including flavivirus group cross-reactive (4G2, 6B3B-3, and 23-2), JE complex cross-reactive (T16 and 7A6C-5), and JEV type-specific (2F2 and 2H4) mAbs.

### Antigen-capture ELISA (Ag-ELISA)

The rabbit anti-JEV polyclonal antibodies were coated on 96-well immunoplates (NUNC) and blocked with StartBlock blocking buffer (Pierce, Rockford, Ill.). JEV or VLP antigens was added into the 96 wells and incubated at 4 °C overnight. After a washing with PBST, the captured JEV or VLP antigens were detected with mouse anti-JEV HIAF or mAbs followed by the addition of peroxidase-conjugated goat anti-mouse IgG (H + L) antibodies (Jackson ImmunoResearch, West Grove, PA). The TMB substrate (Neogen Corp., Lexington, KY) was added into the wells after discarding unbound peroxidase-conjugated antibodies. The reaction was stopped by the addition of 2 N H_2_SO_4_, and the OD_450_ value was read and was used to calculate the endpoint titer of antigens or mAbs by using GraphPad Prism v5.01. The purified GI VLPs were used as the standard to quantify the VLP yield of the clones in Ag-ELISA.

### SDS-PAGE and western blot assay

5X SDS non-reducing sample buffer (315 mM Tris, pH = 6.8, 50% glycerol, 5% SDS, 0.025% bromophenol blue) was prepared and mixed with JEV, VLP antigens, or cellular supernatants. Then, the sample buffer mixed-antigens were denatured in boiling water for 10 min and electrophoretic analyzed using 10% or 12% SDS-PAGE at 80 V for 30 min and 120 V for 70 min. The migrated proteins on gels were transferred to nitrocellulose membranes at 200 V for 3 hrs; membranes were blocked with 5% skimmed milk-PBST and incubated with mouse anti-JEV HIAF, mouse serum specimens, or swine serum specimens at 4 °C overnight. The antibody-bound proteins were detected with peroxidase-conjugated goat anti-mouse IgG (H + L) or goat anti-swine IgG (H + L) antibodies (Jackson ImmunoResearch, West Grove, PA) at 37 °C for 1 hr. The antibody-reactive proteins were visualized by using the LumiGOLD ECL Western Blotting Detection Kit (SignaGen Laboratories, Gaithersburg, MD) with the ImageQuant LAS 4000 mini biomolecular imager (GE Healthcare Life Sciences).

### Transmission electron microscopy

GI JEV and VLPs were concentrated by a 20% sucrose cushion, then purified with 5% to 25% sucrose gradient. Detected by Ag-ELISA, the peak OD_450_ value of infectious virion and GI VLPs in the gradient was further purified by using a centrifugal filter unit (100,000 molecular weight cut-off, Merck Millipore) and recovered in 1X TNE buffer (50 mM Tris, 100 mM NaCl, 0.1 mM EDTA, pH: 7.3). The purified GI JEV and GI JEV VLPs were loaded on carbon-coated copper grids and negatively stained with 2% uranyl acetate. Then, the particles were viewed under the JEOL JEM-1400 Transmission Electron Microscope and recorded by Gatan 895 CCD. These procedures were operated and supported by Dr. SR Wu, National Cheng Kung University, Taiwan.

### Expression, purification, and quantification of GI JEV VLP

The 51-10 clone-produced GI JEV VLPs were continually collected from G418-containing (250 μg in 1 ml) serum-free medium (SFM4MegaVir^TM^, HyClone^TM^) with addition of 1X cholesterol (Gibco) and replaced with the fresh media at 28 °C every 3 days. Cell debris was discarded by centrifuging at 8000 rpm for 30 minutes. The VLP-containing supernatant was primarily purified and concentrated by using a centrifugal filter unit (100,000 molecular weight cut-off, Merck Millipore). Then, the recovered VLPs were further concentrated by 20% sucrose cushion, and purified with 5% to 25% sucrose gradient. The peak OD_450_ value of GI VLPs in the gradient was further purified by using a centrifugal filter unit (100,000 molecular weight cut-off, Merck Millipore) and recovered in 1X PBS. This purified GI JEV VLP was used to immunize mice and swine.

### Vaccination of GI JEV VLPs in mice and swine

Female BALB/c mice — 25 mice (5 mice per group) and 28 mice (20 mice in GI VLP-immunized group and 8 mice in PBS-immunized group) were used for dosage determination and the evaluation of immunogenicity and protective efficacy. The mice at age 6, 8, and 12 weeks were subcutaneously injected with purified GI JEV VLP or PBS mixed with Freund’s incomplete adjuvant (SIGMA-ALDRICH). For swine experiments, 25 and 17 second-generation SPF swine were used for dose determination and the evaluation of immunogenicity and protection efficacy using GI JEV VLPs. These swine at age 4 and 6 weeks received ISA201-adjuvant (SEPPIC) GI JEV VLPs or ISA201-adjuvant (SEPPIC) PBS via the subcutaneous route. We collected blood from mice and swine right before each immunization, and serum specimens were separated by centrifuging and stored at −80 °C.

### Mouse IgG antibody capture (GAC)-ELISA and swine antibody-sandwich ELISA

The endpoint titers of antibody responses among mouse and swine were estimated by the mouse IgG antibody capture (GAC)-ELISA and swine Ag-ELISA as described previously^20,60^. Briefly, goat anti-mouse IgG (H + L) (KPL, Gaithersburg, MD) or serial-diluted swine serum was coated on 96-well immunoplates (NUNC) at 37 °C for 1 hr and blocked with StartBlock blocking buffer (Pierce, Rockford, IL). Then, for mouse GAC-ELISA, the serial diluted mouse serum specimens were added into the wells and incubated at 37 °C for another 90 min. After discarding the un-captured antibodies in the GAC-ELISA and swine antibody-sandwich ELISA, GI WT VLPs and the cellular supernatant were added into the wells and incubated at 4 °C overnight. The next day, the mouse IgG antibody- or swine serum-captured VLPs were recognized by incubating with rabbit or mouse anti-JEV polyclonal antibodies at 37 °C for 1 hr and reacted with peroxidase-conjugated goat anti-rabbit or anti-mouse IgG (H + L) antibodies (Jackson ImmunoResearch, West Grove, PA) at 37 °C for another hour. The steps of coloring and OD detection were the same as for Ag-ELISA. The endpoint titers of mouse IgG antibody and swine serum against VLPs were calculated by the sigmoidal dose–response method in GraphPad Prism v5.01.

### Focus-reduction micro-neutralization titer (FRμNT) assay

FRμNT was used to measure the neutralizing activity of serum specimens. First, 2.48 × 10^4^ VERO cells were seeded on 96-well plates (Corning) and incubated for 18 to 20 hr in a 37 °C incubator supplied with 5% CO_2_. The inactivated serum specimens were serially diluted and incubated with 100 focus forming unit (ffu) of JEV at 37 °C for 1 hr. The VERO cells formed a monolayer and were infected with the serum-virus mixture at 37 °C for 1 hr. The infected cells were overlaid with 1% methyl cellulose and 2% FBS in DMEM and incubated in a 37 °C incubator supplied with 5% CO_2_ for 30 hr. The infected cells were washed with PBS to remove the 1% methyl cellulose, fixed with 75% acetone in PBS at room temperature for 20 min and dried under the hood. The fixed cells were then incubated with mouse anti-JEV HIAF at 37 °C for 40 min, and JEV-reactive mouse antibodies were detected by incubation with peroxidase-conjugated goat anti-mouse IgG (H + L) antibodies (Jackson ImmunoResearch, West Grove, PA) at 37 °C for another 40 min. The virus-infected foci appeared by staining with the Vector-VIP peroxidase substrate kit SK-4600 (Vector Laboratories, Burlingame, CA). The infected cell foci were counted and calculated to determine FRμNT_50_ titers using the sigmoidal dose–response method in GraphPad Prism v5.01. The FRμNT_50_ titers below the cut-off value of 1:10 was arbitrarily given the value of 5 for calculating the geometric mean titer (GMT) of FRμNT_50_ (GMT FRμNT_50_).

### JEV challenge in mice and swine

The GI VLP- and PBS-immunized mice were intracranially injected with 100 LD_50_ GIII and GI JEVs at 2 weeks after the final booster, and survival was recorded until 21 days post-challenge. During this period, mouse was euthanized with CO_2_ as they lost 20% of the initial weight. Immunized swine at age 10 weeks were subcutaneously inoculated with 10^7^ ffu of GIII or GI JEV. The body temperature of experimental swine was monitored daily, and plasma was recovered from sodium citrate treated-blood at 1, 2, 4, 6, 8 days post-challenge. Swine were euthanized on day-8 post challenge. Tonsils, lymph nodes, and brain were harvested after euthanasia. Tissues were cut into two portions for RNA extraction and quantification.

### Micro-plaque assay

This method was used to detect the viral titer which was presented as ffu per ml. The plasma or supernatant from homogenized tonsils was serially diluted and used to infect a monolayer of Vero cells at 37 °C for 1 hr. The same procedures of fixing and staining as described for FRμNT were used.

### Real-time RT PCR

This method was used to quantify JEV RNA in plasma and supernatant from duplicate and homogenized tonsils, lymph nodes, and brain using infectious GI and/or GIII JEV plasmids as the copy-number standard. Total RNA in the cell-mixed plasma or tissues samples was extracted by using the RNeasy Mini kit (Qiagen). vRNAs and cellular mRNAs were reverse transcribed into cDNA with the JEV 3′UTR reverse primer (5′-AGATCCTGTGTTCTTCCTCA-3′) and random primers, respectively, using the SuperScript III Reverse Transcriptase (ThermoFisher). Then, the cDNA transcripts were mixed with JEV 3′UTR primers (5′-TGGGTTAMCAAAGCCGTTGA-3′ and 5′-ACATACTTCGGCGCTCTGTG-3′) or actin primers (5′-TCCTGTGGCATCCACGAAACT-3′ and 5′-GAAGCATTTGCGGTGGACGAT-3′) and the iQ SYBR Green Supermix (Bio-Rad) and amplified for 40 cycles at 95 °C for 10 sec, 65 °C for 30 sec, and 72 °C for 30 sec in the Bio-Rad CFX connect machine (Bio-Rad). Viral RNA quantity was calculated using a full-length cDNA plasmid as the copy-number standard, normalized with actin mRNA by using the Bio-Rad CFX Manager v3.1 (Bio-Rad), and expressed as the copy number per milliliter or per gram.

### Statistical analysis

Student’s two-tailed t-test and one-way ANOVA were used to compare the differences between 2 and multiple groups, respectively. The post-test involved Turkey’s Multiple Comparison Test. These statistical calculations were performed by GraphPad Prism v5.01. P < 0.05 was considered statistically significant.

### Data availability

The datasets generated during and/or analysed during the current study are available from the corresponding author on reasonable request.

### The level of bio-containment

We conducted all JEV-related experiments in a bio-safety level-II facility.

## Electronic supplementary material

Supplementary information
