# Supplementary material for: Genotype I of Japanese Encephalitis Virus Virus-like Particles Elicit Sterilizing Immunity against Genotype I and III Viral Challenge in Swine
Source: Sci Rep. 2018 May 10;8:7481. doi: 10.1038/s41598-018-25596-1 (PMC5945781; doi:10.1038/s41598-018-25596-1)
Supplement: Supplementary file 1 — Supplementary information [file 41598_2018_25596_MOESM1_ESM.pdf]

Supplementary Information

Genotype I of Japanese Encephalitis Virus Virus-like Particles Elicit Sterilizing Immunity against Genotype I and III Viral Challenge in Swine

Yi-Chin Fan, Jo-Mei Chen, Jen-Wei Lin, Yi-Ying Chen, Guan-Hong Wu, Kuan-Hsuan Su, Ming-Tang Chiou, Shang-Rung Wu, Ji-Hang Yin, Jiunn-Wang Liao, Gwong-Jen J. Chang & Shyan-Song Chiou

Protein expression and secretion of GI JEV VLP-expressing plasmids. VLP is a by-product secreted from JEV infected cells, and is secreted from cells transfected with the plasmid encoding viral premembrane (prM) and envelope (E) genes of GIII JEV[1]. Thus, we constructed a GI JEV VLP-expressing plasmid encoding prM and E genes of GI JEV, namely pVJGI YL2009-4 WT (Supplementary Fig. 1A), and plasmid-transfected COS-1 cells were analyzed by indirect immunoflorescence assay (IFA) using mouse anti-JEV hyperimmune ascitic fluid (HIAF). The signal of VLP antigens were observed in the pVJGI YL2009-4 WT-transfected cells but not in pVAX control-transfected cells (Supplementary Fig. 1B). After establishing a cell clone that continually expressed GI VLP, we constructed another GI VLP-expressing plasmid carrying the neomycin

resistance gene named pcDNAJGI YL2009-4 WT, which was derived from pVJGI YL2009-4 WT (Supplementary Fig. 1C). We measured the VLP antigens being expressed and the secretion of pcDNAJGI YL2009-4 WT in heparan sulfate-deficient CHO-pgsA745 cells (CHO-HS(-)), which are low susceptible to JEV infection[2]. The positive signal of VLP antigens was shown in the pcDNAJGI YL2009-4 WT-transfected CHO-HS(-) cells (Supplementary Fig. 1D). Then, we harvested VLP antigens from the day-old culture media of the plasmid-transfected COS-1 and CHO-HS(-) cells, and the end-point VLP titer was determined by the antigen-capture ELISA (Ag-ELISA) with titers in the range of 38 to 75 and 30 to 58, respectively. We compared and examined the viral proteins expressed by COS-1 and CHO-HS(-) cells-derived VLP and JEV-infected culture supernatants by electrophoresis and Western blotting. The viral E, NS1, prM, and M proteins were detectable in JEV sample, and the same size of E and prM proteins were in the VLPs secreted from either COS-1 or CHO-HS(-) cells (Supplementary Fig. 1E). These results indicated VLPs were successfully translated and secreted from pVJGI YL2009-4 WT- and pcDNAJGI YL2009-4 WT-transfected cells.

Establishment of the CHO-HS(-) clone to consistently produce GI JEV VLPs. The use of transiently transfected cells to produce a VLP vaccine requires purified plasmids

and a cumbersome process to separate dead and non-antigen producing cells. We selected and established a VLP-expressing CHO-HS(-) cell line, which might reduce the re-attachment of the secreted VLPs on the heparan sulfate-deficient cell surface[2]. Thus, VLPs expressed in this cell line may reduce the cell fusion and improve the production of GI JEV VLPs. The pcDNAJGI YL2009-4 WT-transfected and non-transfected CHO-HS(-) cells were cultured with G418, and cell viability was recorded under light microscopy at 0, 5, and 12 days post-treatment (DPT) (Supplementary Fig. S3). The non-transfected CHO-HS(-) cells became rounded and detached from the flask at 5 DPT, with complete cell death at 12 DPT. However, a portion of pcDNAJGI YL2009-4 WT-transformed cells survived at 12 DPT (Supplementary Fig. S3). The G418 resistant GI plasmid-transformed cells were sub-cloned twice using the limited dilution method in 96-well plates, and cell clones secreted VLP antigens were detected by Ag-ELISA. Ten VLP-positive clones secreted VLPs with the Ag-ELISA titers ≥ 128. Five 100% IFA positive cell clones (4-4, 51-2, 51-3, 51-6, and 51-10). The 4-4, 51-6, and 51-10 were selected to determine the stability of VLP secretion (Supplementary Fig. S4A).

The dynamic yield of VLP antigens secreted from the selected clones were estimated every day for 6 days (Supplementary Fig. S4B). The VLPs were detectable at day 1, reached a plateau at day 3 and decreased after day 4. The 51-6 and 51-10

clones produced a higher amount of VLP, 702.7 and 968.9 ng/ml, respectively. Then, we evaluated the stability of VLP expression by three clones by passaging cells every three days for 10 passages. The 51-6 and 51-10 clones constantly secreted VLP antigens for 10 passages, with an average of 475.1 and 820.4 ng/ml. In contrast, the VLP yield in 4-4 clone was reduced after the 5th passage (Supplementary Fig. S4C). Immunofluorescent assay results showed 100% maintenance of VLP-positive cells of 51-6 and 51-10 clones but only half the positive cells of the 4-4 clone at passage 10 (Supplementary Fig. S4D). The 51-10 clone was selected, because of its better stability and efficiency in secreting VLPs to produce GI JEV VLP for the following study.

Supplementary Methods

Construction of GI JEV VLP-expressing plasmids. pVJGI YL2009-4 WT and pcDNAJGI YL2009-4 WT were GI JEV VLP-expressing plasmids used in the study and encoded genes in order as human cytomegalovirus early gene promoter (CMV IE), kozak sequence (5’-GCCGCCGCC-3’), the modified JE signal sequence (5’-ATGGGCAAGAGGTCCGCCGGCTCAATCATGTGGCTCGCGAGCTTGGCAGTTGTCATAGCTGGTACAAGCGCT-3’), prM (nucleotide position 478 to 978 in the genome of GI YL2009-4 strain), 80% E genes (nucleotide position 979 to 2175 in the genome of GI YL2009-4 strain) and 20% E genes (nucleotide position 2175 to 2477 in the genome of GIII SA14 strain, KU323483.1) with chimeric human β-globin gene intron (5’-GTAAGTATCAAGGTTACAAGACAGGTTTAAGGAGACCAATAGAAACTGGGCTTGTCGAGACAGAGAAGACTCTTGCGTTTCTGATAGGCACCTATTGGTCTTACTGACATCCACTTTGCCTTTCTCTCCACAG-3’), and bovine growth hormone poly(A) signal. First, the pVJGI YL2009-4 WT was constructed by replacing prM and 80% E genes of the dengue serotype I virus (DENV1) VLP-expressing plasmid (pV8D1-2J-i)[5] with the corresponding region of GI YL2009-4 strain in the reaction of In-Fusion PCR cloning kit (Clontech, USA). Briefly, prM and 80% E genes of GI YL2009-4 strain were amplified from viral cDNA with the forward (5’-

ATAGCTGGTACAAGCGCTATGAAGCTATCAAACTTTCAAGG-3’) and the reverse (5’-GCCTTGCCCAGCGTGCTTCCAGCTTTGTGCCAGTGATGGTTAATC-3’) primers in PCR reaction of Q5® High-Fidelity 2X Master Mix (New England Biolabs, USA). The remaining sequences of pVJGI YL2009-4 WT were amplified from pV8D1-2J-i with the forward (5’ GGAAGCACGCTGGGCAAGGC-3’) and reverse primers (5’- AGCGCTTGTACCAGCTATGAC-3’). The two PCR products were annealed in the reaction of In-Fusion PCR cloning kit following the manufacturers protocols. The final product of the cloning kit was transformed into competent cells, and it recovered the pVJGI YL2009-4 WT clone. Then, the modified JE signal sequence, prM, and E genes of pVJGI YL2009-4 WT were transferred to pcDNA3.1 with KpnI and NotI restriction enzymes to recover the pcDNAJGI YL2009-4 WT carrying the Neomycin resistant gene.

Selection of the stable cell line secreting GI JEV VLP. GI JEV VLPs were produced from the stable clone transformed with the pcDNAJGI YL2009-4 WT plasmid. The pcDNAJGI YL2009-4 WT plasmid was transfected into CHO-HS(-) cells by using lipofectamine 2000 (Invitrogen) following the manufacturers protocols. The next day, the culture media was replaced with F12 medium supplemented with 10% heat-

inactivated FBS and 500 μg Geneticin (G418, ThermoFisher) in 1 ml and incubated in the 37°C incubator until observing death of 90% of cells among the non-transfected CHO-HS(-) cells. After G418 treatment, surviving cells among the pcDNAJGI YL2009-4 WT-transformed CHO-HS(-) cells underwent limiting dilution twice. The single and stable clone was evaluated with the ability and stability of VLP secretion by the yield of VLP and maintenance of JEV-antigen positive cells after ten passages of the clone.

Indirect immunoflorescence assay (IFA). Cells transformed with VLP-expressing plasmids or control vectors were seeded on Millicell EZ SLIDE 8-well glass plates (Merck Millipore). The next day, the transformed cells were fixed with 4% paraformaldehyde (Sigma-Aldrich) in PBS (AppliChem Panreac), permeated with 0.05% TritonX 100 (Sigma-Aldrich) in PBS, and then blocked with 5% skimmed milk in PBS. After blocking, the cells expressing JEV antigens were detected with mouse anti-JEV HIAF followed by the addition of anti-mouse IgG (H+L) antibodies conjugated with Alex488 (KPL, Gaithersburg, MD). The cell nucleus was located by staining with DAPI (Invitrogen). Images were viewed and recorded by fluorescence microscopy using OLYMPUS CKX41.

Histopathology. The brain specimens were harvested from JEV challenged pigs and fixed with 10% of formalin (Fisher Scientific). The fixed brain samples were trimmed and embedded in paraffin. Then, the paraffin sample block was cut into 2 μm thickness and stained by hematoxylin and eosin (H&E). The histopathological change was observed and recorded by a light microscopy (BX-51, Olympus Tokyo, Japan).

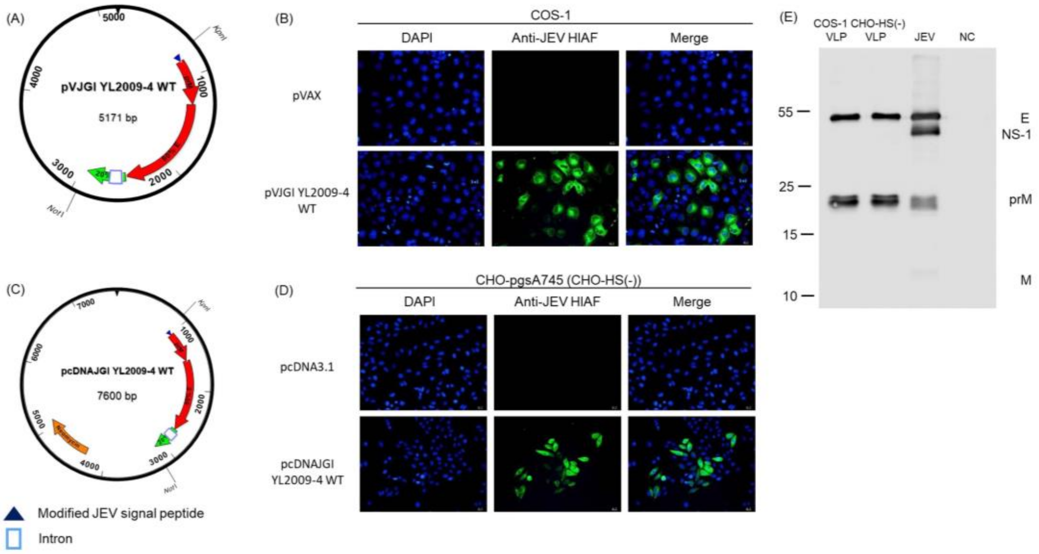

Supplementary Figure S1. Characterization of GI JEV VLP-expressing plasmids. The prM and 80% of E genes (ectodomain region) encoded by GI JEV YL2009-4, and 20% of the E gene (transmembrane region) of the GIII SA-14 strain[3] were incorporated into pVAX and pcDNA3.1 vectors to establish the plasmids expressing GI JEV VLP, called pVJGI YL2009-4 WT (A) and pcDNAJGI YL2009-4 WT (C). The modified JE signal peptide sequence was located in front of the prM gene, and the intron was inserted within the end of the 20% E gene [4]. The GI VLP antigen was detected in pVJGI YL2009-4 WT-transformed COS-1 cells (B) and pcDNAJGI YL2009-4 WT-transformed CHO-pgsA745 (CHO-HS(-)) cells (D) by IFA with mouse anti-JEV hyperimmune acetic fluid (anti-JEV HIAF) staining. The pVAX- and pcDNA3.1-

transfected cells were a negative control (NC). (E) The secreted VLP antigens were harvested and analyzed by Western blot assay stained with anti-JEV HIAF antibody. GI JEV VLP antigens collected from the supernatant of transfected COS-1 or CHO-HS(-) cells are indicated as COS-1 VLP or CHO-HS(-) VLP. The supernatant collected from GI JE YL2009-4 virus-infected or vector-transfected cells are presented as JEV or NC. Full-length Western blots are presented in Supplementary Fig. S2.

Supplementary Figure S2. Full-length Western blots for Supplementary Figure 1E and Figure 1B.

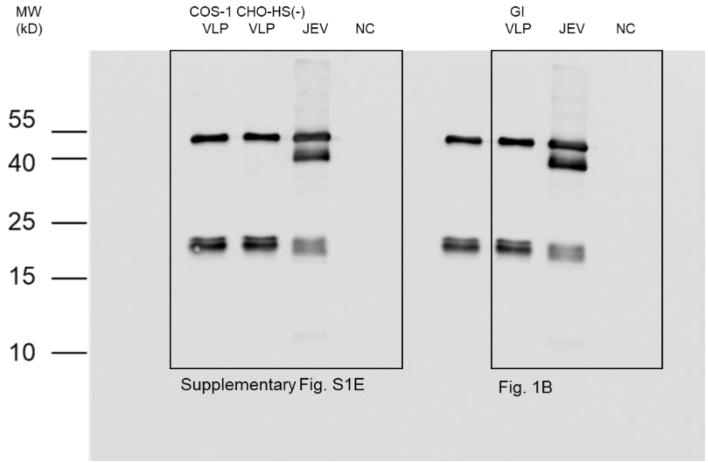

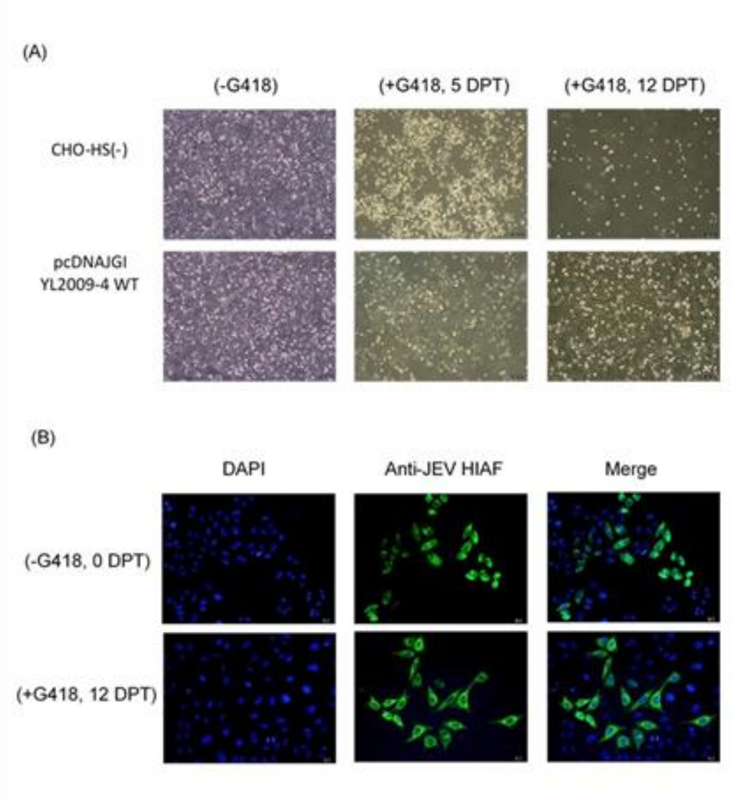

Supplementary Figure S3. G418 selection of the CHO-HS(-) cell-derived stable clones expressing GI JEV VLP. (A) The viability of pcDNAJGI YL2009-4 WT-transformed or non-transformed CHO-HS(-) cells was recorded at day 1, 5, and 12 post-G418 treatment by light microscopy. (B) Before the limiting dilution, JE signals were detected in plasmid-transformed cells that were untreated (-G418, 0 DPT) or treated (+G418, 12 DPT) with G418 by IFA.

(A)

Clones[a]	VLP secretion[b]	Positive cells[c] (%)
4-1	16	93
4-4	64	100
4-6	16	96
51-2	128	100
51-3	128	100
51-6	128	100
51-10	256	100
53-1	128	69
53-2	128	64
53-5	64	58
53-6	128	75
53-12	128	91
62-7	128	57
62-9	128	54
62-12	16	20

[a] Clones were selected after G418 treatment followed by the limiting dilution
[b] VLP secretion was indicated as the endpoint titer of Ag-ELISA
[c] Positive cells expressing JEV antigens were calculated in IFA

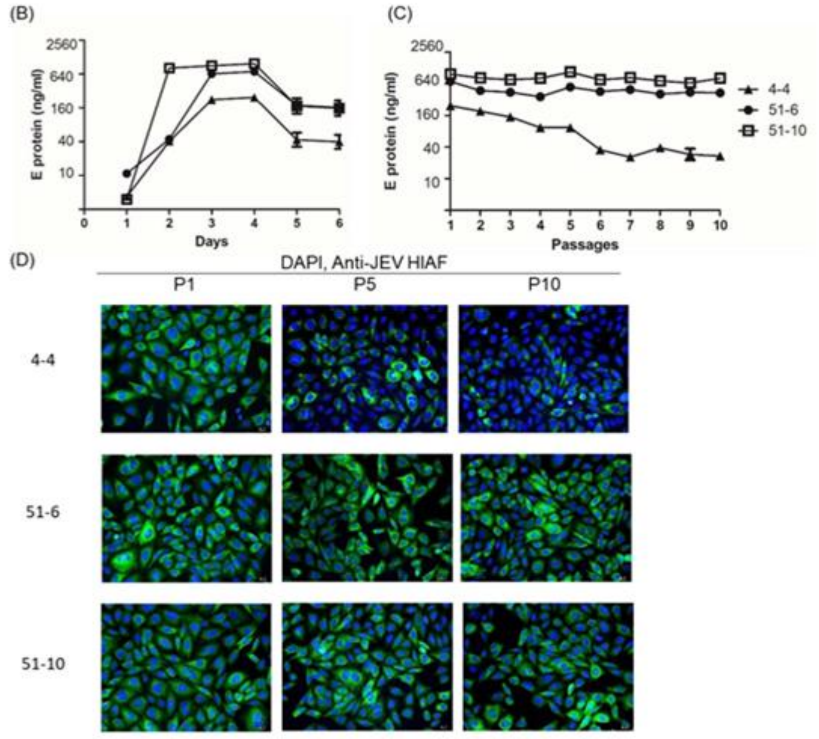

Supplementary Figure S4. Establishment of the GI VLP-expressing CHO-HS(-) stable clones. (A) After incubation with G418 for 12 days, surviving pcDNAJGI YL2009-4 WT-transformed CHO-HS(-) cells were limiting diluted and cultured twice. The single clone was selected and VLP secretion was measured by Ag-ELISA and JEV-positive rate by IFA. (B) The dynamic yield of VLP antigens secreted from the selected 4-4, 51-6, and 51-10 clones was estimated every day for 6 days. (C) The stability of 4-4, 51-6, and 51-10 clones on VLP expression was evaluated by passaging every 3 days for 10 passages. The yield was estimated by Ag-ELISA by using the standard of purified GI VLP and presented as the concentration of E protein (nanograms per ml). (D) The

positive rate of JEV signal was determined among the 4-4, 51-6, and 51-10 clones at passage 1 (P1), 5 (P5), and 10 (P10) by IFA.

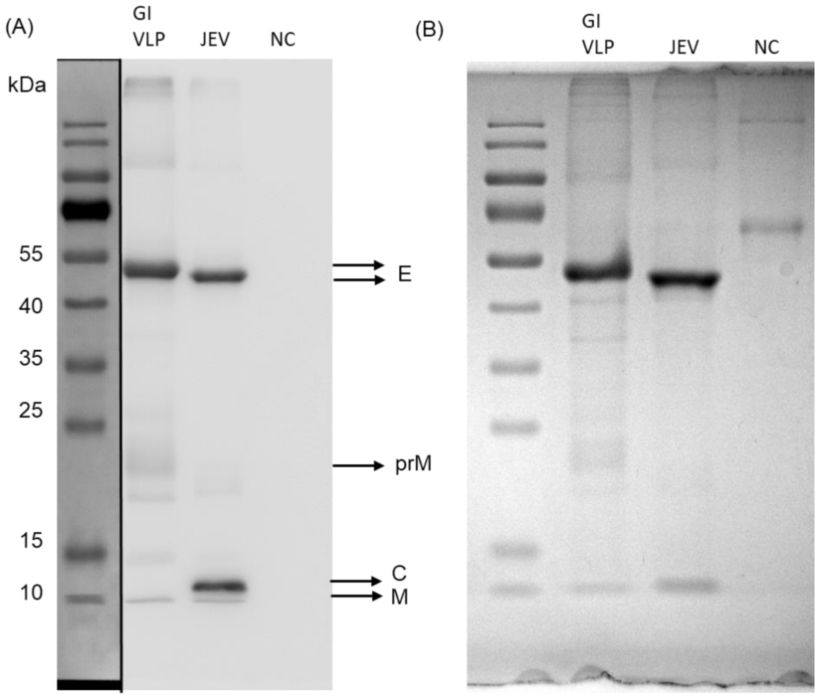

Supplementary Figure S5. The CHO-HS(-) derived 51-10 clone-produced and purified GI JEV VLP sample was analyzed by Western blot assay (A) with anti-JEV HIAF and is indicated as GI VLPs. Total protein of the GI JEV VLP-purified sample was examined in Coomassie blue-stained SDS page (B). The supernatant collected from GI JE YL2009-4 virus-infected or the non-infected C6/36 cells (*Aedes albopictus* clone) is presented as JEV or NC (negative control).

190 **Supplementary Figure S6. Full-length Western blots for Figure 2C**

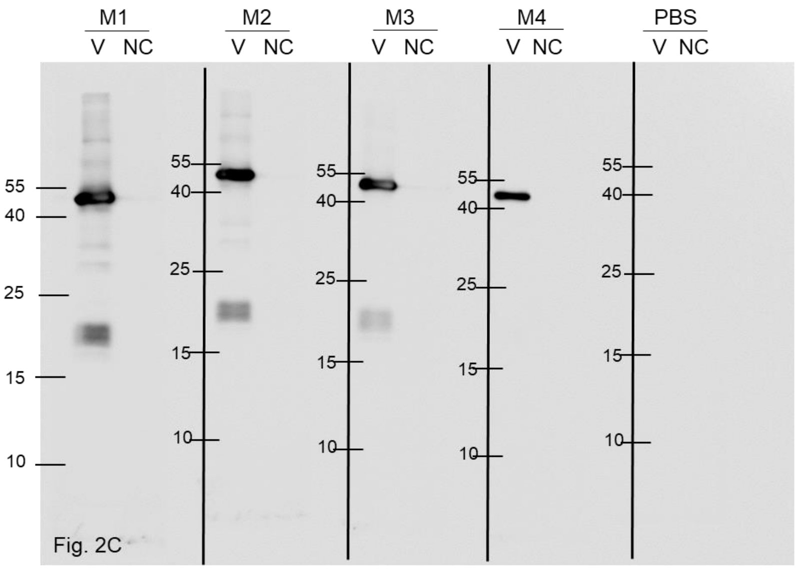

191

192

193 **Supplementary Figure S7. Full-length Western blots for Figure 3C**

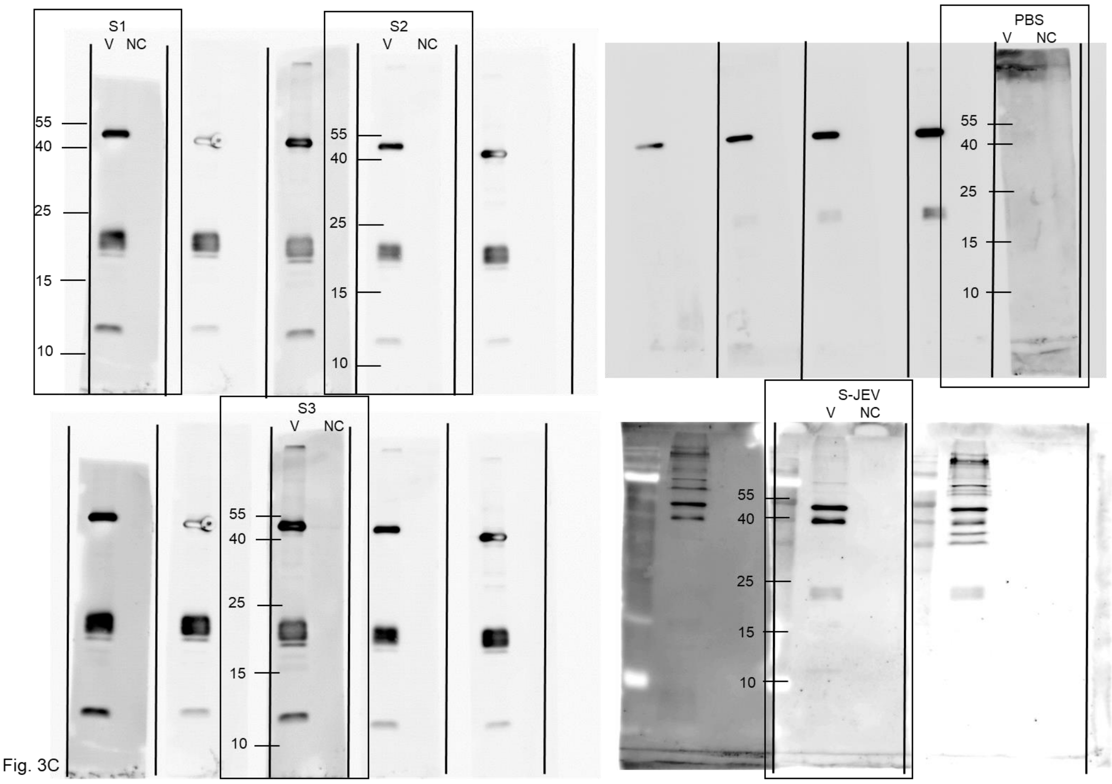

194

195

196

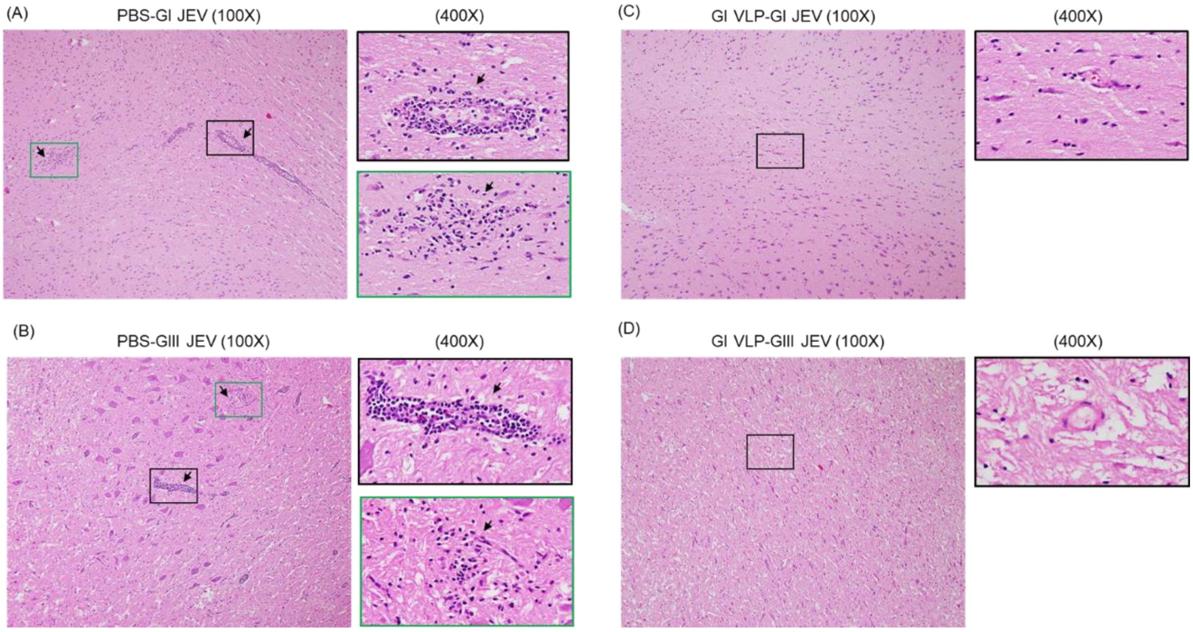

Supplementary Figure S8. Histopathological changes of immunized SPF pig brain after JEV challenge. Immunized pigs at age 10 weeks were subcutaneously injected with 10^7 ffu JEVs. Brains were harvested from PBS-immunized pigs (A and B) and GI VLP-immunized pigs (C and D) at 8 days after challenge with GI YL2009-4 virus (A and C) or GIII CH1392 virus (B and D). Histology was examined by hematoxylin and eosin staining under low magnification (100X) and high magnification (400X). Multifocal lymphocytic perivascular cuffing and gliosis are indicated with arrows and highlighted with black and green rectangles, respectively.

Supplementary References

1 Chang, G. J., Hunt, A. R. & Davis, B. A single intramuscular injection of recombinant plasmid DNA induces protective immunity and prevents Japanese encephalitis in mice. *Journal of virology* **74**, 4244-4252 (2000).

2 Chien, Y. J., Chen, W. J., Hsu, W. L. & Chiou, S. S. Bovine lactoferrin inhibits Japanese encephalitis virus by binding to heparan sulfate and receptor for low density lipoprotein. *Virology* **379**, 143-151, doi:10.1016/j.virol.2008.06.017 (2008).

3 Purdy, D. E. & Chang, G. J. Secretion of noninfectious dengue virus-like particles and identification of amino acids in the stem region involved in intracellular retention of envelope protein. *Virology* **333**, 239-250, doi:10.1016/j.virol.2004.12.036 (2005).

4 Chiou, S. S., Fan, Y. C., Crill, W. D., Chang, R. Y. & Chang, G. J. Mutation analysis of the cross-reactive epitopes of Japanese encephalitis virus envelope glycoprotein. *The Journal of general virology* **93**, 1185-1192, doi:10.1099/vir.0.040238-0 (2012).

5 Crill, W. D. *et al.* Sculpting humoral immunity through dengue vaccination to enhance protective immunity. *Frontiers in immunology* **3**, 334, doi:10.3389/fimmu.2012.00334 (2012).
